# Hydrogen Peroxide and Dopamine Sensors Based on Electrodeposition of Reduced Graphene Oxide/Silver Nanoparticles

**DOI:** 10.3390/s24020355

**Published:** 2024-01-07

**Authors:** Yuhang Zhang, Na Li, Bo Liu, Hangyu Zhang

**Affiliations:** 1 School of Biomedical Engineering, Faculty of Medicine, Dalian University of Technology, Dalian 116024, China; 32009288@mail.dlut.edu.cn (Y.Z.); lina316@dlut.edu.cn (N.L.); lbo@dlut.edu.cn (B.L.); 2 Liaoning Key Lab of Integrated Circuit and Biomedical Electronic System, Dalian University of Technology, Dalian 116024, China

**Keywords:** silver nanoparticles, reduced graphene oxide, electrochemical sensors, dopamine, hydrogen peroxide

## Abstract

In this work, silver nanoparticles (AgNPs)/reduced graphene oxide (rGO) nanocomposites were electrodeposited on glassy carbon electrodes (GCE) to construct electrochemical sensors for the detection of hydrogen peroxide (H_2_O_2_) and dopamine (DA). The AgNPs were synthesized on graphene oxide (GO) by the hydrothermal method, followed by the reduction of the GO during the electrodeposition process, resulting in the formation of the nanocomposites on the surface of the electrodes. The generation of AgNPs on the graphene sheets was verified by scanning electron microscopy (SEM) and electrochemical impedance spectroscopy (EIS). The AgNPs/rGO/GCE showed a linear response to H_2_O_2_ in the range of 5 μM to 620 μM, with a sensitivity of 49 μA mM^−1^cm^−2^ and a limit of detection (LOD) of 3.19 μA. The linear response of the AgNPs/rGO/GCE to DA ranged from 1 μM to 276 μM, the sensitivity was 7.86 μA mM^−1^cm^−2^, and the LOD was 0.18 μM. Furthermore, DA and H_2_O_2_ were detected simultaneously in the same solution without interferences, and the sensors displayed good stability over time. The preparation method for the sensors is relatively eco-friendly, convenient, and efficient, exhibiting great potential for sensitive detection of DA and H_2_O_2_.

## 1. Introduction

Electrochemical biosensors can be divided into enzymatic and non-enzymatic biosensors. Enzymes are proteins or RNAs produced by living cells with high specificity and catalytic efficiency for their substrates. Therefore, enzyme-based biosensors are usually highly selective and sensitive. However, since the catalytic performance of an enzyme depends on its own spatial structure, changes in the external environment may lead to decreased efficiency of enzymatic biosensors due to enzyme inactivation. Consequently, non-enzymatic electrochemical sensors have received considerable attention in practical applications [1]. In recent years, nanocomposites using graphene as the electrochemical substrate and metal nanoparticles to provide catalytic activity have also attracted much attention in the field of non-enzymatic electrochemical sensors.

Graphene, a single-atom-thick planar sheet composed of sp2-bonded carbon atoms, has attracted much attention due to its unique electrical, mechanical, thermal, and optical properties, thereby holding great promise in many advanced technologies, such as nanoelectronics, sensors, capacitors, and composites [2,3,4,5,6,7,8,9]. The favorable properties of graphene, such as high conductivity, a large surface-to-volume ratio, and excellent chemical resistance, make it an attractive substrate for composite materials. Accordingly, metal nanoparticle-decorated graphene composites have become the focus of research in recent years. Silver nanoparticle-modified graphene composites, in particular, have been extensively studied due to their effectiveness in a range of applications, including surface-enhanced Raman scattering substrates, catalysis, and antibacterial properties [10,11,12,13].

H_2_O_2_ is a widely used oxidant that can be produced as a by-product of most oxidases. It is widely used as a disinfectant in different chemical industries and an essential intermediate in many biological reactions, so its detection is receiving increasing attention [14,15,16]. Therefore, there is great interest in the fabrication of cost-effective, rapid, selective, sensitive, and stable H_2_O_2_ sensors. A number of techniques have been designed to estimate H_2_O_2_ concentrations, but most have disadvantages, including their requiring expensive instrumentation, low selectivity, low sensitivity, or being time-consuming [17,18,19].

DA is a major catecholamine neurotransmitter that plays a vital role in the cardiovascular and central nervous systems. The concentration level of DA in the body can affect the physical health status of people. High DA levels can lead to cardiotoxicity, further leading to increased heart rate, hypertension, and heart failure [20]. In contrast, low levels of DA in the central nervous system are thought to be a major cause of several neurological disorders, such as Parkinson’s disease, schizophrenia, and Alzheimer’s disease [21]. The level of dopamine in the human body is affected by the concentration of dopamine precursors, so the detection of dopamine precursors such as phenylalanine and tyrosine has become popular [22,23,24]. Direct measurement of DA levels is also crucial for understanding the biological functions and processes involved.

Hydrogen peroxide and dopamine are commonly used analytes in biosensor technology because of their importance in their respective fields. Silver nanomaterials are widely used in electrochemical detection, and recently Rayhane et al. successfully fabricated H_2_O_2_ sensors using prepared silver nanoplate [25]. However, compared with the metal particles themselves, GO and silver nanoparticles are more widely used because GO can provide abundant growth sites for metal nanoparticles, and their complexes are more stable. Wang et al. used a one-pot method in 2013 to prepare nano-silver-modified rGO for H_2_O_2_ detection with good sensitivity [26]. In 2015, Yang et al. synthesized a nanocomposite AgNPs–Tween–GO where TWEEN80 was employed as a modifier of the GO and a stabilizer of the AgNPs. Possibly due to the participation of the TWEEN80, which resulted in the average size and better stability of the silver nanoparticles, the modified electrode obtained by simple drop addition had a linear response to H_2_O_2_ in the range of 0.02 to 23.1 mM. One downside to this approach was that it necessitated specific limiting conditions of high pressure and high temperature during the preparation of the silver nanoparticles [27]. In a recent study, AgNPs were immobilized on the surface of MoS2–GO to selectively detect DA in the presence of uric acid and ascorbic acid without reporting the continuous detection performance and repeatability [28].

Here, we report a rapid and eco-friendly approach for the fabrication of AgNPs/rGO-based biosensors. The AgNPs/GO was first prepared using the hydrothermal method, and the growth of the AgNPs on graphene sheets was examined by SEM and EIS. The AgNPs/GO was converted to AgNPs/rGO by electrodeposition and modified on the surface of the electrode to prepare the sensor. The sensor can detect DA and H_2_O_2_, with the sensitivity, detection limit, and linear range being tested by electrochemical methods. This facile, fast, and eco-friendly methodology produced sensors that were highly sensitive and stable for DA and H_2_O_2_ detection.

## 2. Materials and Methods

### 2.1. Materials and Reagents

GO solution (GO, 1.27 wt%) was purchased from Chengdu Organic Chemicals Co., Ltd. (Chengdu, China). Anhydrous sodium citrate (C_5_H_5_O_7_Na_3_, ≥99%), NaCl (99.5%), and silver nitrate solution (AgNO_3_, 0.5 M) were purchased from Shanghai Aladdin Biochemical Technology Co., Ltd. (Shanghai, China). Hydrogen peroxide (H_2_O_2_, 30%) was purchased from Tianjin Kemiou Chemical Reagent Co., Ltd. (Tianjin, China). Dopamine hydrochloride (DA, 98%), KCl (99.5%), glucose (99%), and ethanol (C_2_H_6_O, 99.7%) were purchased from Shanghai Macklin Biochemical Technology Co., Ltd. (Shanghai, China). Phosphate buffer solution (PBS) was purchased from Beijing Solarbo Science & Technology Co., Ltd. (Beijing, China). Uric Acid (UA, 98%) was purchased from Bide Pharmatech Co., Ltd. (Shanghai, China). 

### 2.2. Apparatus

CV and amperometric measurements were performed on a CHI1040C electrochemical workstation (Shanghai Chenhua Co. Ltd., China). EIS experiments were performed on a CHI660E electrochemical workstation (Shanghai Chenhua Co., Ltd., Shanghai, China). All the electrochemical experiments were performed using a standard three-electrode system consisting of a 3 mm diameter glassy carbon electrode as the working electrode, an Ag/AgCl electrode as the reference electrode, and a Pt wire electrode as the counter electrode. The SEM was undertaken using a NOVA NanoSEM 450 for the characterization of surface morphology. The AgNPs/GO was characterized using an X-ray diffractometer (XRD; D/MAX-2400, Rigaku, The Woodlands, TX, USA) and a UV-Vis spectrophotometer (Evolution 201, Thermo Scientific, Waltham, MA, USA).

### 2.3. Preparation of AgNPs/GO Composites

The AgNPs/GO was synthesized according to a reported method with slight modifications [29]. Briefly, 100 μL of 5 mM AgNO_3_ was mixed with 300 μL of 12.7 mg/mL GO in 2.6 mL ultrapure water. The solution was then ultrasonically dispersed for half an hour. Afterward, the mixture was added to 17 mL ultrapure water, which had been heated to 60 °C and stirred magnetically for 4 h. The mixed solution was magnetically stirred at 60 °C for 2 h after adding 14.8 mg of sodium citrate. Subsequently, the AgNPs/GO composite solution was obtained.

### 2.4. Preparation of AgNPs/rGO Modified GCE

The GCE (3 mm in diameter) was polished with 1.0, 0.3, and 0.05 µm alumina powders on a polishing cloth and rinsed with deionized water, followed by sonication in ethanol and deionized water, in turn, and allowed to dry at room temperature. The polished electrode should not be left exposed to air for an extended period. The polished glassy carbon electrode was placed in 5 mL of an AgNPs/rGO complex solution and electrodeposited with a standard three-electrode system at a voltage of −1.3 V for 600 s to obtain AgNPs/rGO/GCE. For comparison, GO/GCE was prepared by drop addition, while rGO/GCE was prepared by electrodeposition.

## 3. Results

### 3.1. The Growth of AgNPs on GO

**Figure 1:**
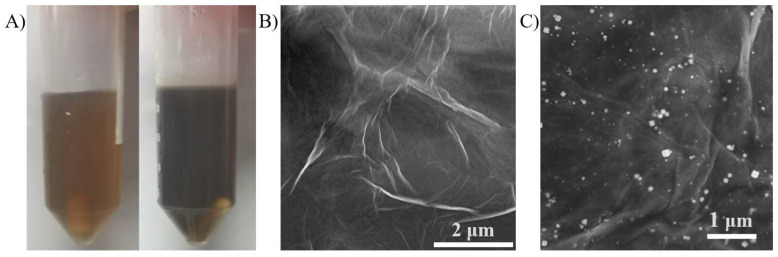
The growth of AgNPs on GO. (**A**) The photo of the mixed solution of AgNO_3_ and GO before and after heating. (**B**) The SEM image of rGO. (**C**) The SEM image of AgNPs/rGO.

Figure 1A shows the photos of a mixed solution of AgNO_3_ and GO containing sodium citrate before and after heating. It can be seen that the color of the mixed solution of AgNO_3_ and GO changed from light yellow to brown-black after heating. The change in the color of the solution indicated that the composition of the solution had changed. To further demonstrate the growth of AgNPs on the surface of graphene sheets and to analyze the microstructure characteristics of the AgNPs/rGO composites, the AgNPs/rGO composites were electrodeposited on glassy carbon sheets and examined by SEM. For comparison, the glassy carbon sheet modified by GO was prepared using the drop addition method. The sheet structure with wrinkles that illustrated the fundamental feature of graphene was observed in both the rGO and AgNPs/rGO samples (Figure 1B,C). This suggested that the preparation process of AgNPs/rGO did not damage the GO structure. Moreover, the growth of AgNPs on the graphene sheets was observed, demonstrating the successful electrodeposition of AgNPs/rGO on the glassy carbon surface (Figure 1C).

**Figure 2:**
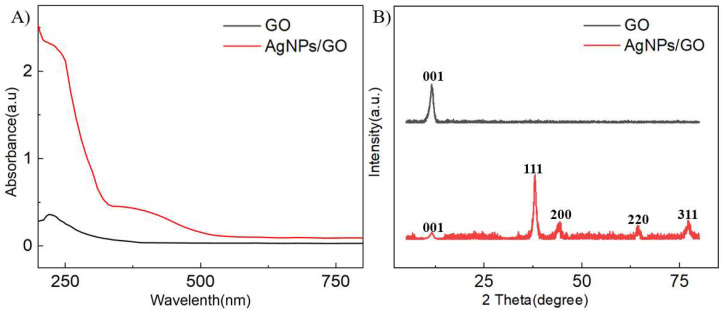
Structure characterization of AgNPs/GO. (**A**) The absorbance spectra of GO and AgNPs/GO. (**B**) The XRD spectra of GO and AgNPs/GO.

To further explore the composition of the complex, UV-Vis and XRD analysis of the complex solution were performed. As shown in Figure 2A, the GO and AgNPs/GO were characterized by UV-Vis spectroscopy. The GO exhibited a characteristic peak at around 230 nm, corresponding to the π-π* transition of C=C [30]. The observation of a shoulder peak at 230 nm in the AgNPs/GO sample indicated that the GO structure remained intact during the AgNPs growth. Furthermore, the AgNPs/GO also displayed a peak at approximately 427 nm, which accounted for the characteristic surface plasmon resonance of the AgNPs with λ_max_ within 400–500 nm [31]. XRD was applied to analyze the characteristics of the GO and AgNPs/GO powder samples, including the crystalline phase, morphology, and microstructure. Figure 2B depicts the typical characteristic (002) peak at 10.81° of GO. Compared with the GO, the (002) peak of the AgNPs/GO displayed a decreasing trend, attributable to the growth of AgNPs on the surface of the graphene nanosheets, which hindered the re-stacking of the graphene nanoplates [32,33]. Moreover, the AgNPs/GO had newly indexed peaks at 38.1°, 44.3°, 64.4°, and 77.4°, reflecting the cubic phase of Ag (PDF card number: 00-001-1167). The results of the UV-Vis and XRD showed that AgNPs were successfully grown on the surface of the graphene sheet, and the 2D nanostructure of GO was still present.

### 3.2. Electrochemical Characterization of AgNPs/rGO/GCE

**Figure 3:**
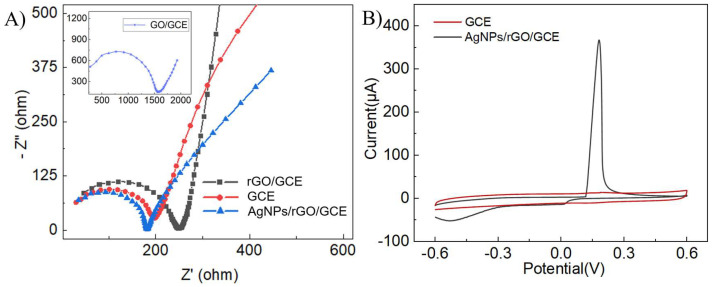
Electrochemical characterization of AgNPs/rGO/GCE. (**A**) EIS curves of GCE, GO/GCE, rGO/GCE, and AgNPs/rGO/GCE. (**B**) The CV curves of GCE and AgNPs/rGO/GCE in PBS.

The electrochemical properties of the surface-modified GCE were analyzed using EIS, performed in 20 mM [Fe(CN)_6_]^3−/4−^, 0.1 M KCl over a frequency range of 10^6^ Hz to 10 Hz. Nyquist plots of GCE, GO/GCE, rGO/GCE, and AgNPs/rGO/GCE are shown in Figure 3A, where the diameter of the semicircle indicates the charge transfer resistance (R_ct_). The rise in R_ct_ after GO modification (GO/GCE) on the surface of the GCE could be attributed to decreased conductivity caused by excessive oxygen-containing groups on the surface of the GO. Therefore, after the electric reduction of the GO (rGO/GCE), the reduction of oxygen-containing groups on the surface led to a sharp increase in the overall conductivity of the electrode and a decrease in R_ct_. The growth of AgNPs on the graphene sheet increased the specific surface area of the electrode and promoted electron transfer, thereby lowering the R_ct_ of AgNPs/rGO/GCE. The analysis of AgNPs/rGO/GCE, rGO/GCE, GO/GCE, and GCE by EIS indicated that AgNPs grew on the graphene and that AgNPs/rGO/GCE was successfully electrodeposited on the surface of the GCE.

CV tests were conducted on the modified electrodes to further investigate the electrochemical properties of AgNPs/rGO/GCE. When immersed in PBS and subjected to CV scanning at a voltage range spanning from −0.5 V to 0.4 V, the AgNPs/rGO/GCE exhibited an oxidation peak at 0.15 V, which represented the characteristic peak of silver nanoparticles, thereby demonstrating the growth of AgNPs on the GO surface. Moreover, it could be seen that the AgNPs/rGO/GCE had a tiny reduction peak at 0 V, indicating the reduction peak of Ag_2_O. Additionally, the reduction peak at −0.4 V might correspond to the reaction of oxygen in the solution catalyzed by AgNPs. The obtained CV curves remained stable over 30 cycles (Figure S1), suggesting the good electrochemical stability of AgNPs/rGO/GCE.

### 3.3. The Changes in Specific Surface Area

To analyze the changes in the specific surface area of the GCE after modification with AgNPs/rGO, the CV of the GCE and AgNPs/rGO/GCE were tested in a solution containing 20 mM K_3_[Fe(CN)_6_], 0.1M KCl, from −0.2 V to 0.8 V, with the scanning speed varied from 0.02 V/s to 0.2 V/s. With the Randles–Sevcik equation, $Ip=\left(2.69\times 10^{5}\right)n^{\frac{3}{2}}AD^{\frac{1}{2}}V^{\frac{1}{2}}C$, the specific surface area of each electrode was calculated. The specific surface area of the modified material is measured in A/(cm^2^), while the concentration of the redox mediator is given in C/(mol·cm^−3^). The cyclic voltammetry rate is represented as V/(V · S^−1^), and D/(cm^2^/s) is the diffusion coefficient. The peak current is Ip/(A), and n refers to the number of transferred electrons. The diffusion coefficient, D, of K_3_[Fe(CN)_6_] is 7.6 × 10^−6^ cm^2^s^−1^. The detailed CV test results at varying scanning speeds of the GCE and AgNPs/rGO/GCE are shown in Figures S2 and S3. The CV peak current of the AgNPs/rGO/GCE was observed to be greater than that of the GCE. The peak currents were fitted to the scan rate to obtain Figure S4. The electroactive area of the AgNPs/rGO/GCE was calculated to be 24.8 mm^2^.

Figure S5A shows the CV curves of the AgNPs/rGO/GCE at different scan rates. PBS was chosen as the supporting electrolyte to obtain the maximum sensitivity of the sensor. As can be seen from the figure, the potential and peak current depend on the scan rate. The peak cathode current increased linearly as the scan rate increased from 20 to 200 mV s^−1^, with a correlation coefficient of 0.973 (Figure S5B), indicating that the redox process of the fabricated bio-nanocomposites was a surface-controlled process.

### 3.4. The Detection of H_2_O_2_ by AgNPs/rGO/GCE

Based on the previously reported mechanism [34,35,36], the electrocatalytic reaction of H_2_O_2_ on the electrocatalyst occurred according to the following mechanism:

$$H_{2}O_{2}+e^{-}\overset{\;AgNPs\;}{\to }OH_{ads}+OH^{-}$$

$$OH_{ads}+H^{+}+e^{-}\overset{\;\;AgNPs\;\;}{\to }H_{2}O$$

According to the first equation, the H_2_O_2_ that was adsorbed on the AgNPs gained an electron, producing (OH)_ads_ and OH^−^. Subsequently, the (OH)_ads_ received another electron from the AgNPs and produced H_2_O. The reduction rate here depended mainly on the adsorption of H_2_O_2_ on the electrocatalyst surface and the transfer of electrons from the electrocatalyst to (OH)_ads_. Therefore, the enhanced adsorption and electron transfer properties of the electrocatalyst are necessary for the electrocatalytic reduction of H_2_O_2_.

**Figure 4:**
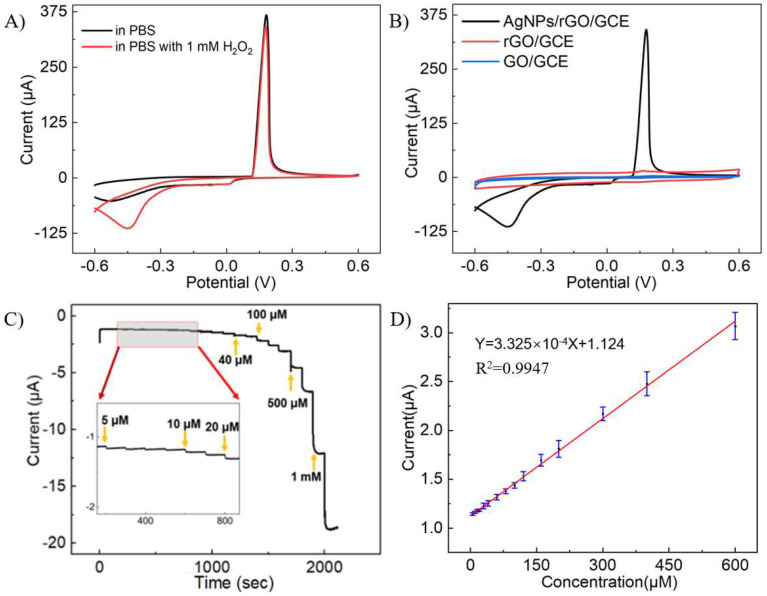
CV responses of different electrodes and amperometric tests of AgNPs/rGO/GCE to H_2_O_2_. (**A**) CV curves of AgNPs/rGO/GCE in PBS solution with or without 1 mM H_2_O_2_. (**B**) CV curves of AgNPs/rGO/GCE, rGO/GCE, and GO/GCE in PBS solution with 1 mM H_2_O_2_. (**C**) Current responses at -0.3 V of AgNPs/rGO/GCE in PBS with different concentrations of H_2_O_2_. (**D**) The calibration curve of the current responses in (**C**).

To verify the catalytic effect of AgNPs on H_2_O_2_ reduction, CV tests were conducted on GO/GCE, rGO/GCE, and AgNPs/rGO/GCE in PBS solution containing 1 mM H_2_O_2_. Upon the addition of 1 mM H_2_O_2_, there was a significant current peak at around −0.35 V, accounting for H_2_O_2_ reduction catalyzed by AgNPs compared with AgNPs/rGO/GCE in PBS solution without H_2_O_2_ (Figure 4A). On the contrary, GO/GCE and rGO/GCE barely responded to H_2_O_2_, as illustrated in Figure 4B. This indicated that the presence of AgNPs facilitated the transfer of electrons to H_2_O_2_ during the electrocatalytic reduction. The role of AgNPs in this process was to increase the specific surface area of the electrode and catalyze the reaction of H_2_O_2_, which enhances the electron transfer kinetics during its electrocatalytic reduction. Upon successively introducing H_2_O_2_ into the system, the amperometric responses of the AgNPs/rGO/GCE were examined at −0.3 V. The AgNPs/rGO/GCE displayed responses to H_2_O_2_ in a wide range, from 5 μM to 3.62 mM, and a response time (t_95_) of around 3 s (Figure 4C). The response current increased linearly with H_2_O_2_ concentration between 5 μM and 620 μM, with an R^2^ of 0.998. The sensitivity was calculated to be 49 μA mM^−1^cm^−1^, and the LOD was estimated to be 3.19 μM (S/N = 3).

### 3.5. The Detection of DA by AgNPs/rGO/GCE

**Figure 5:**
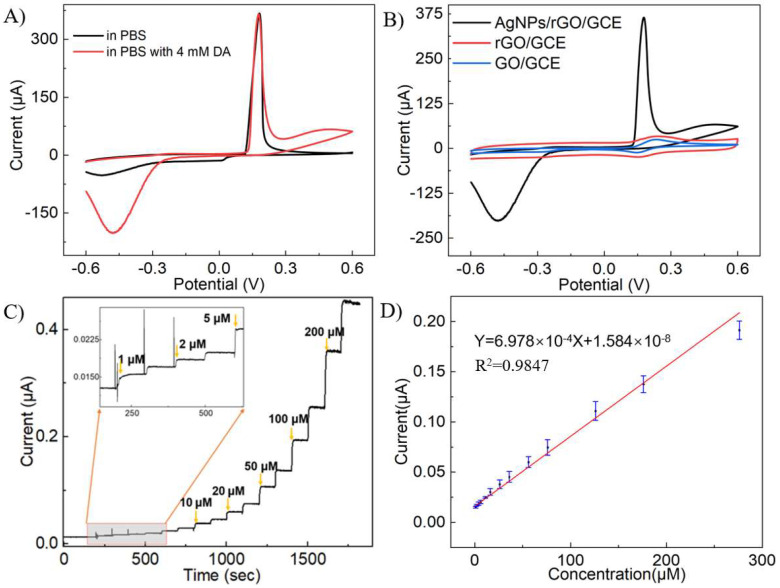
CV responses of different electrodes and amperometric tests of AgNPs/rGO/GCE to DA. (**A**) CV curves of AgNPs/rGO/GCE in PBS with or without 4 mM DA. (**B**) CV curves of AgNPs/rGO/GCE, rGO/GCE, and GO/GCE in PBS solution with 4 mM DA. (**C**) Current responses at -0.3 V of AgNPs/rGO/GCE in PBS with different concentrations of H_2_O_2_. (**D**) The calibration curve of the current responses in (**C**).

CV measurements with a voltage spanning from −0.6 V to 0.6 V and a scanning rate of 100 mV·s^−1^ were conducted on AgNPs/rGO/GCE in PBS solution containing 4 mM DA to explore the optimal detection voltage for DA. Upon adding 4 mM DA, there was an oxidation peak at 0.5 V and a reduction peak at −0.45 V, indicating that the AgNPs catalyzed DA reduction in comparison to AgNPs/rGO/GCE in a PBS solution lacking DA (Figure 5A). In contrast, GO/GCE and rGO/GCE showed minimal response to DA, as illustrated in Figure 5B.

In order to have a better signal-to-noise ratio of the system, the amperometric test was conducted at a voltage of 0.25 V. Figure 5C illustrates the current changes in the system when various concentrations of DA were added, and the AgNPs/rGO/GCE responded to the DA in a wide range, from 1 μM to 776 μM. It was observed that the current response swiftly rose to a stable level within 3 s after adding DA, suggesting a quick response rate of the system. Figure 5D exhibits the curve fitting of the response current to the concentration. The linear response range of the system was from 1 μM to 276 μM (R^2^ = 0.997). The sensitivity was calculated as 7.86 μA mM^−1^cm^−1^, and the LOD for DA was estimated to be 0.18 μM (S/N = 3).

The CV curves of AgNPs/rGO/GCE responded at both 0.5 V and −0.45 V after the addition of DA. However, the AgNPs/rGO/GCE had no obvious current response when the amperometric tests were applied to detect DA at any voltage ranging from −0.2V to −0.6V. In terms of the report from Chen et al., AgNPs-modified GCE would have a reduction peak from −0.4 V to −0.6 V in the oxygenated solution [37]. Therefore, it was speculated that when DA was added, oxygen catalyzed the reaction of DA, resulting in a reduction peak of CV at this voltage. However, the oxygen in the solution would soon be exhausted, as amperometric tests would take a long period, and as a result, the reduction peak was unsuitable for monitoring DA in the amperometric tests.

Table 1 and Table 2 show the sensors used to detect H_2_O_2_ and DA in recent years. The sensor performance prepared in this paper was comparable to the sensors previously reported, and the preparation process was convenient, green, and fast. It is worth mentioning that Golsheikh et al. electrodeposited AgNPs-rGO onto an ITO electrode surface in one step by applying CV to the mixed solution of [Ag(NH_3_)_2_OH] and GO in 2013 [37]. Their method of preparing the sensor was also very convenient, but the LOD of the prepared sensor was high.

### 3.6. Interference and Stability

**Figure 6:**
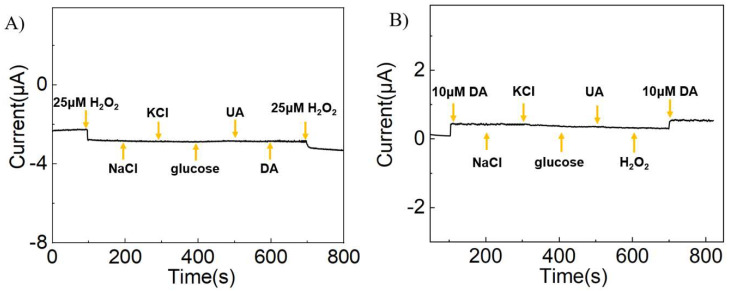
Selectivity test. (**A**) The current response of AgNPs/rGO/GCE was obtained by adding 25 μM H_2_O_2_, 0.1 M NaCl, 0.1 M KCl, 0.1 M glucose, 0.1 M UA, 0.1 M DA, and 25 μM H_2_O_2_ to PBS solution at a voltage of −0.3 V. (**B**) The current response of AgNPs/rGO/GCE was obtained by adding 10 μM DA, 0.1 M NaCl, 0.1 M KCl, 0.1 M glucose, 0.1 M UA, 0.1 M H_2_O_2_, and 10 μM DA to PBS solution at a voltage of 0.25 V.

To investigate the anti-interference performance of AgNPs/rGO/GCE on detecting DA and H_2_O_2_ simultaneously, amperometric tests were conducted at 0.25 V and −0.3 V, respectively, and the results are shown in Figure 6. The AgNPs/rGO/GCE displayed negligible current response after the addition of NaCl, KCl, glucose, and UA compared with the sharp response to H_2_O_2_ at −0.3 V or to DA at 0.25 V. Importantly, the presence of H_2_O_2_ or DA did not interfere with the detection of the other.

**Figure 7:**
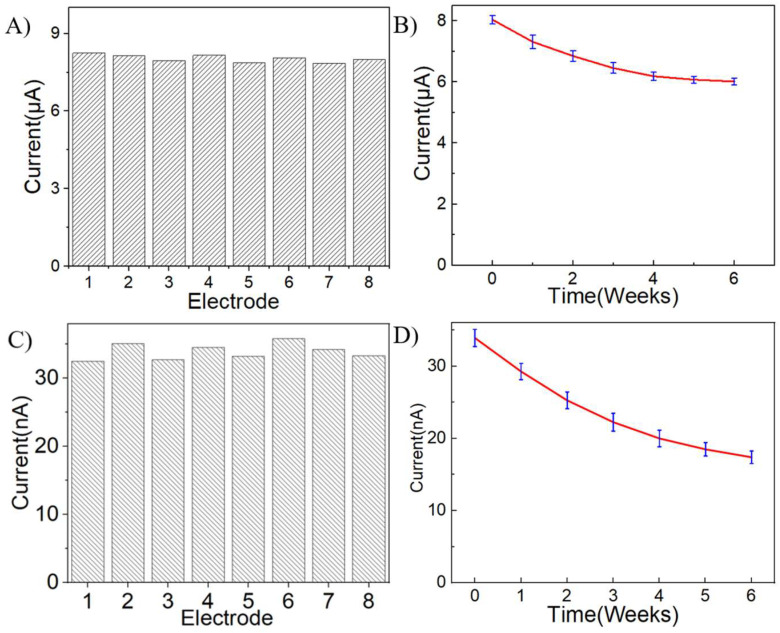
Stability test. (**A**) Peak currents of eight different AgNPs/rGO/GCEs in PBS solution containing 1 mM H_2_O_2_. (**B**) Changes in the peak current of oxidation in a PBS solution containing 1 mM H_2_O_2_ for eight different electrodes after 1–6 weeks in the refrigerator. (**C**) Peak currents of eight different AgNPs/rGO/GCEs in PBS solution containing 10 μM DA. (**D**) Changes in the peak current of oxidation in a PBS solution containing 10 μM DA for eight different electrodes after 1–6 weeks in the refrigerator.

To demonstrate the stability and utility of the method, we also evaluated the precision and long-term stability of the AgNPs/rGO/GCE. Eight sensors modified with AgNPs/rGO by the same method were prepared, and the peak currents of CV in PBS solution containing 1 mM H_2_O_2_ for each electrode were recorded. As shown in Figure 7A, a relative standard deviation of 1.7% for the eight electrodes was obtained, indicating a high reproducibility of our method for preparing sensor electrodes. In addition, these electrodes were used to measure the peak current at weekly intervals for 6 weeks to test the long-term stability. When not in use, the electrodes were immersed in PBS and placed at 4 °C. The sensitivity still maintained around 75% of the initial value after 6 weeks (Figure 7B), demonstrating good stability of the sensors. Similar results were obtained for DA detection using AgNPs/rGO/GCE (Figure 7C,D). The relative standard deviation of DA detection was 1.81%. After 6 weeks, the current response of the sensors to DA remained at 62.4% of the initial value.

## 4. Conclusions

In this work, AgNPs/GO composites were prepared in solution, followed by the electrodeposition of AgNPs/rGO on the surface of GCE to obtain AgNPs/rGO/GCE. The modification of AgNPs/rGO on GCE effectively increased the specific surface area of the GCE and gave the GCE the capacity to respond to H_2_O_2_ and DA, respectively. The sensors exhibited high sensitivity, a wide linear range, low detection limit, excellent reproducibility, and relatively good long-term stability for both H_2_O_2_ and DA. Besides, the AgNPs/rGO/GCE responded to H_2_O_2_ and DA at different voltages and could detect them simultaneously. This convenient and eco-friendly method might have great potential for sensor preparation for multiple target detection.

## Figures and Tables

**Table 1:** A comparison of this work with works in the literature regarding the performance of the H_2_O_2_ assays.

Sensor for H_2_O_2_	Linear Range (mM)	Detection Limit (μM)
GQD-PNF-GO [38]	0.01–7.2	0.055
AgNPs-rGO(one-pot) [26]	0.05–5	10
AgNPs/PDA/rGO [39]	0.005–9.97	0.68
3D-rGO/AgNP [40]	0.016–27	6.8
Ag/ZIF–8 [41]	0.02–5, 5.5–10	6.2
N–graphene–AgND [42]	0.1–80	0.26
Ag nanowire array [43]	0.1–3.1	29.2
ERGO-Ag [44]	0.1–100	1.6
AgNPs/rGO [this work]	0.005–0.62	3.19

**Table 2:** A comparison of this work with works in the literature regarding the performance of the DA assays.

Sensor for DA	Linear Range (μM)	Detection Limit (μM)
N-rGO [45]	1–60	0.1
PEDOT-GO [46]	6–200	2.0
rGO-Ag/PANI [47]	5–200	0.2
MoS_2_/Ag [48]	1–500	0.2
MoS_2_/rGO/AgNP [28]	2.5–12.5	0.009
AgNPs/rGO [this work]	1–276	0.18

## Data Availability

Data are contained within the article and Supplementary Materials.

## Supplementary Materials

The following supporting information can be downloaded at: https://www.mdpi.com/article/10.3390/s24020355/s1.
