# Supplementary material for: Hydrogen Peroxide and Dopamine Sensors Based on Electrodeposition of Reduced Graphene Oxide/Silver Nanoparticles
Source: Sensors (Basel). 2024 Jan 7;24(2):355. doi: 10.3390/s24020355 (PMC10818837; doi:10.3390/s24020355)
Supplement: Supplementary file 1 [file sensors-24-00355-s001.zip › sensors-2755494-supplementary.pdf]

Supporting information

Hydrogen peroxide and dopamine sensors based on electrodeposition of reduced graphene oxide/silver nanoparticles

Yuhang Zhang [1], Na Li [1,2] Bo Liu [1,2] and Hangyu Zhang [1,2,*]

[1] School of Biomedical Engineering, Faculty of Medicine, Dalian University of Technology, Dalian 116024, China
[2] Liaoning Key Lab of Integrated Circuit and Biomedical Electronic System, Dalian University of Technology, Dalian 116024, China
* Correspondence: hangyuz@dlut.edu.cn

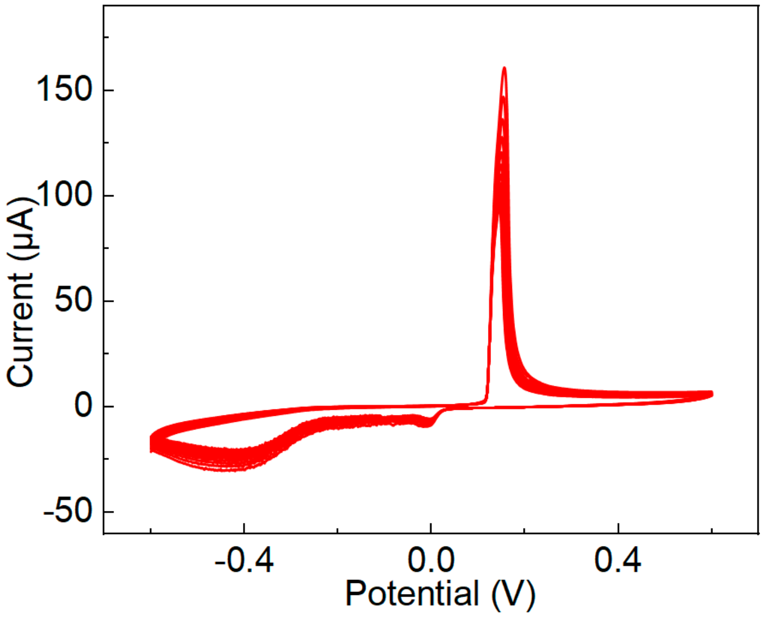

Figure S1. CV curves of AgNPs/rGO/GCE electrodes scanned for 30 cycles in PBS solution.

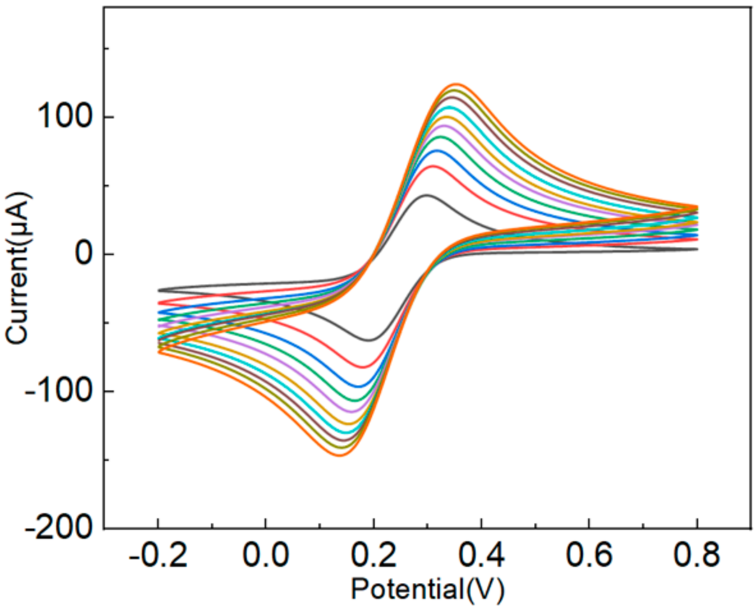

Figure S2. CV curves of the GCE in 20 mM $K_3[Fe(CN)_6]$, 0.1 M KCl solution with scanning speed from 0.02 V/S to 0.2 V/S.

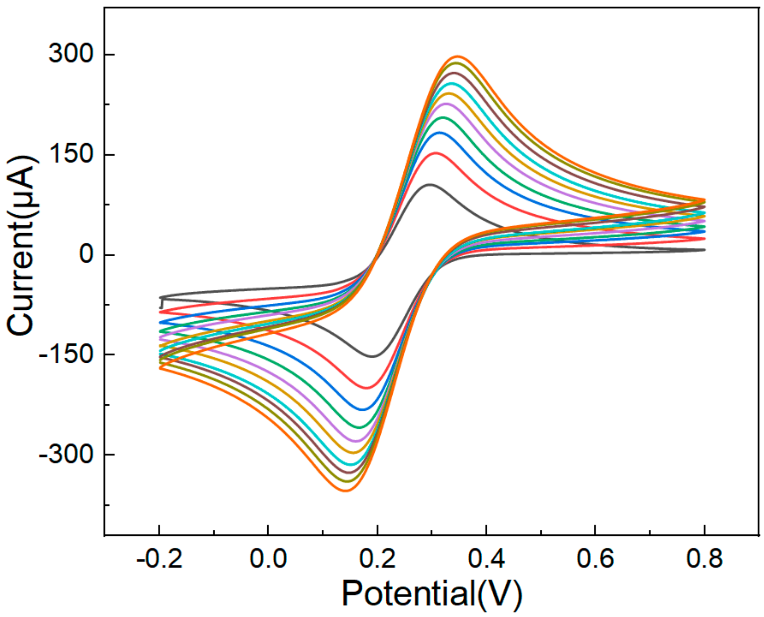

Figure S3. CV curves of the AgNPs/rGO/GCE in 20 mM $K_3[Fe(CN)_6]$, 0.1 M KCl solution with scanning speed from 0.02 V/S to 0.2 V/S.

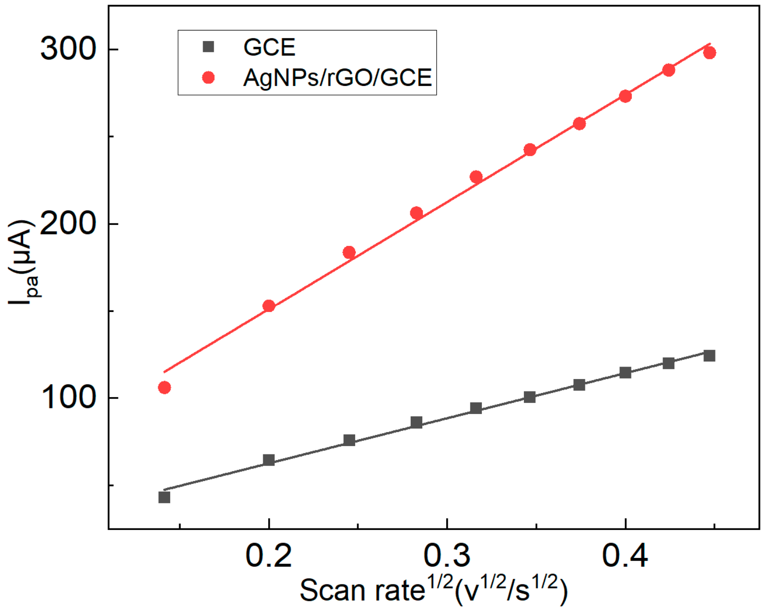

Figure S4. Fitting curves of the peak current values of GCE and AgNPs/rGO/GCE vs. the scanning speed.

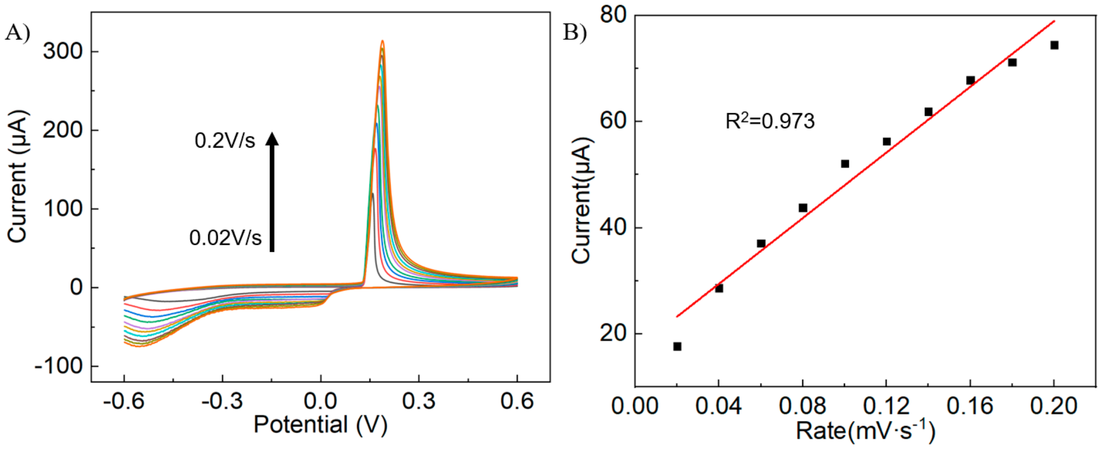

PFigure S5. (**A**) CV curves of AgNPs/rGO/GCE in PBS solution at different scan rates from 20 to 200 mV/s. (**B**) Plots of cathodic peak currents vs. scan rate.
